# The Transformative Role of Molecular, Cellular, and Blood Biomarkers in Precision Medicine

**DOI:** 10.3390/biom15121680

**Published:** 2025-12-02

**Authors:** Jordi Camps, Isabel Fort-Gallifa, Xavier Gabaldó-Barrios

**Affiliations:** 1 Unitat de Recerca Biomèdica, Hospital Universitari Sant Joan, Institut d’Investigació Sanitària Pere Virgili, Universitat Rovira i Virgili, Av. Dr. Josep Laporte 2, 43204 Reus, Spain; 2 Department of Clinical Laboratory, Hospital Universitari Joan XXIII, Institut d’Investigació Sanitària Pere Virgili, Universitat Rovira i Virgili, C. Dr. Mallafré Guasch 4, 43005 Tarragona, Spain; isabel.fort@urv.cat; 3 Oxidative Stress Commission of SemedLab, C. Padilla 323, 08025 Barcelona, Spain; 4 Department of Clinical Laboratory and Autoimmunity, Infection and Thrombosis Research Group (GRAIIT), Department of Internal Medicine, Hospital Universitari de Sant Joan, Institut d’Investigació Sanitària Pere Virgili, Universitat Rovira i Virgili, Av. Dr. Josep Laporte 2, 43204 Reus, Spain; xavier.gabaldo@salutsantjoan.cat

## 1. The Expanding Role of Biomarkers in Modern Medicine

The importance of molecular, cellular, and blood-based biomarkers in modern medicine cannot be overstated. These tools are reshaping how diseases are detected, monitored, and treated, and the contributions gathered in this Special Issue illustrate their growing relevance in the study of oncology, cardiovascular disease, and neurodegeneration [1]. As healthcare moves toward increasingly personalized approaches, biomarkers provide essential insights into disease mechanisms and support the development of more precise and effective therapies.

Early detection remains one of the strongest motivations for biomarker research. Identifying diseases before symptoms appear can dramatically improve survival and quality of life. This is particularly evident in conditions where clinical signs emerge at advanced stages, such as lung cancer. Likewise, the introduction of plasma phosphorylated tau 217 (p-tau217) as a marker for the preclinical stages of Alzheimer’s disease demonstrates how accessible biomarkers can expand early diagnostic capabilities. Because amyloid-β pathology develops years before cognitive decline, reliable blood-based indicators, such as p-tau217, have the potential to guide interventions when treatments are most likely to be effective [2,3]. Early detection transforms care from reactive to preventive, enabling timely therapies and targeted follow-up in high-risk individuals.

Equally important is the prognostic value. Understanding how a disease is likely to progress is crucial for guiding treatment decisions and predicting patient outcomes. Prognostic markers can provide valuable insights into the likelihood of recurrence, metastasis, or disease progression, informing therapy choices. In oncology, for instance, tumor-specific markers help clinicians predict how a patient will respond to treatments like immunotherapy or targeted therapy [4]. The ability to anticipate treatment responses is essential for selecting the most effective therapies and minimizing side effects. The relevance of prognostic biomarkers extends far beyond cancer, encompassing other major chronic diseases associated with aging, such as cardiovascular and neurodegenerative disorders [5].

Personalized medicine, often referred to as precision medicine, is another area in which biological indicators are making a profound impact. Instead of assuming uniform responses to therapy, precision medicine accounts for the molecular profile of each patient to select the most appropriate treatment. This strategy increases therapeutic success and reduces adverse effects by avoiding ineffective interventions. Classic examples, such as HER2 expression in breast cancer guiding trastuzumab therapy, illustrate how molecular profiles can determine whether a given treatment is truly justified [6].

Beyond guiding therapy selection, biomarkers are also indispensable for monitoring disease progression and treatment efficacy. With the rise of complex cancer therapies such as immunotherapy and targeted therapy, real-time indicators provide crucial information about how patients respond to treatment. For example, liquid biopsies that detect tumor DNA in blood offer a non-invasive means to track tumor dynamics and adjust therapy accordingly [7]. This continuous monitoring is particularly vital in diseases like cancer, where rapid changes in disease status require swift therapeutic decisions.

The utility of these indices extends beyond individual patient care into public health. They can reveal patterns in disease prevalence, risk factors, and population health trends. Screening programs incorporating validated indices help identify at-risk groups who may benefit from early interventions, lifestyle modifications, or prophylactic treatments, ultimately reducing the overall burden of disease [8].

However, despite the promise of biomarkers, their implementation in clinical practice is not without challenges. One significant hurdle is the need for rigorous validation. While the term biomarker is widely used in the scientific community, it is essential to emphasize that not all measurable parameters qualify as such. To be considered a true marker, a parameter must distinctly differentiate between well-defined biological states, such as health versus disease or responsiveness versus resistance to treatment, while minimizing overlap. Achieving such high levels of sensitivity and specificity, especially in complex diseases like cancer and metabolic disorders, remains an ongoing challenge [9].

On the other hand, while blood-based biomarkers offer substantial convenience, their ability to capture the full complexity of disease processes is limited. Complementary approaches, such as imaging or tissue biopsies, are often required to provide a more comprehensive view of disease status. Standardization is another critical issue. Reproducible and comparable results require harmonized protocols across laboratories and healthcare systems. International testing standards are needed to ensure that biomarker-based diagnostics are dependable in real-world settings [10].

Furthermore, integrating these technologies with bioinformatics and artificial intelligence is crucial to enhancing their clinical utility. The vast datasets generated by omic analyses, when coupled with machine learning and deep learning tools, can accelerate the discovery of novel indicators and reveal previously undetected disease signatures. Artificial intelligence-driven algorithms can also assist in interpreting complex data, helping clinicians make faster and more accurate decisions. As these technologies evolve, the potential for discovering new, more precise tools will continue to grow, offering even greater promise for disease management.

Another challenge that often goes underreported is the high cost and the sophisticated analytical methods required for these tools. Many of these advanced diagnostic and prognostic methods rely on complex technologies that are expensive and resource-intensive, making them difficult to implement on a wide scale. The translation of these methods from research settings into routine clinical practice remains a significant barrier, particularly in lower-resource environments. Reducing costs and streamlining these processes will be key to ensuring that these innovations are accessible to all patients, not just those in specialized centers. Moreover, the use of biomarkers in precision medicine raises ethical concerns about privacy, consent, and potential discrimination based on genetic or molecular profiles. These issues need to be carefully considered and addressed to ensure the responsible and equitable use of biomarkers in healthcare.

Looking ahead, multi-biomarker panels are expected to gain prominence. Many diseases, particularly cancer and chronic inflammatory or neurodegenerative conditions, are too heterogeneous to be fully characterized by a single marker. Panels combining complementary indicators provide a more comprehensive view of disease biology and allow for more accurate diagnosis, prognosis, and treatment monitoring. Emerging biomarker families continue to broaden the range of potential applications, opening new opportunities for clinical translation and personalized healthcare [11,12].

In this context, the articles included in this Special Issue provide a representative overview of these advances, illustrating how different types of biomarkers are being applied to specific diseases and offering an up-to-date perspective on their clinical potential.

## 2. An Overview of Published Articles

### 2.1. Original Articles

Murcia-Mejía et al. (contribution 1) explored the metabolic profiles of non-small cell lung cancer patients before and after conventionally fractionated radiation therapy (CFRT) or stereotactic body radiation therapy (SBRT). Semi-targeted metabolomics revealed 32 elevated and 18 reduced metabolites in patients compared with healthy controls, with discriminatory metabolites including ethylmalonic acid, taurine, glutamic acid, maltose, glycocolic acid, and d-arabinose. Importantly, the metabolic changes following CFRT and SBRT differed, reflecting the distinct therapeutic impacts of each approach. By combining metabolomics with machine learning, the authors identified signatures capable of perfectly discriminating patients from controls. This study underscores the potential of metabolic biomarkers not only for early diagnosis but also for monitoring therapeutic response, offering a powerful example of precision oncology in practice.

Kozłowski et al. (contribution 2) assessed serum cytokines and related proteins as potential diagnostic biomarkers for endometrial cancer. Several cytokines distinguished endometrial cancer patients from those with noncancerous lesions with promising sensitivity and specificity. Their findings suggest that panels of immune-related biomarkers could enhance early detection and offer a cost-effective, minimally invasive approach for screening, while also providing a foundation for future studies exploring prognostic and predictive utility.

Middlezong et al. (contribution 3) introduced a novel amplification-free nanopore sequencing approach, CRISPR/Cas9-enriched nanopore sequencing with adaptive sampling (CENAS), for rapid detection of PML::RARA fusions in acute promyelocytic leukemia. CENAS enabled fast, low-cost, point-of-care detection of both typical and atypical fusion breakpoints, with concordance to standard diagnostics. This method exemplifies how genomic biomarkers can facilitate immediate treatment initiation, potentially reducing early mortality and providing insights into fusion-associated pathophysiology.

Hegyi et al. (contribution 4) examined tumor HLA class I and II expression and immune cell infiltration in melanoma patients undergoing PD-1 inhibitor therapy. The study demonstrated that higher HLA-II expression, combined with HLA-I/II scores, and increased immune cell infiltration correlated with treatment response and progression-free survival. These results highlight the predictive value of immunological biomarkers in stratifying patients for immunotherapy and guiding personalized treatment decisions.

Xin et al. (contribution 5) profiled circulating non-coding RNAs in Alzheimer’s disease (AD), identifying dysregulated lncRNAs, piRNAs, and snoRNAs with potential as diagnostic biomarkers. Using machine learning approaches, the authors highlighted specific ncRNA signatures capable of distinguishing AD patients from controls with high accuracy. This study emphasizes the promise of plasma-based RNA biomarkers and machine learning tools for early, non-invasive detection of neurodegenerative diseases.

Wang et al. (contribution 6) dissected the interaction between a pyroptosis-inducing tetranectin epitope and a monoclonal antibody. By identifying two critical contact residues required for binding, the study refines the molecular understanding of this biomarker and guides the design of synthetic antibodies. Such work exemplifies how structural biomarker studies can inform both diagnostics and therapeutic development in sepsis.

Sprenzel et al. (contribution 7) analyzed neutrophil surface glycosylation in horses with recurrent uveitis, a model for human autoimmune disease. They observed increased O-glycosylation levels in diseased animals and identified Integrin beta-2 and CUB domain-containing protein 1 as potential anchoring proteins. This research highlights glycan modifications as functional biomarkers of immune activation, with potential relevance for early detection and monitoring of autoimmune disorders.

### 2.2. Review Articles

Quarta et al. (contribution 8) reviewed circulating biomarkers associated with coronary microvascular disease (CMD), including markers of endothelial dysfunction, inflammation, and oxidative stress. They highlighted the utility of non-invasive blood tests to complement invasive assessments, offering insights into pathophysiology, early diagnosis, and risk stratification. Such biomarkers could enhance clinical management of CMD, a frequently underdiagnosed condition.

Pawluk et al. (contribution 9) focused on the pleiotropic cytokine IL-6 in ischemic stroke. Elevated IL-6 levels were found to be implicated in oxidative stress, vascular injury, and leukocyte recruitment. The review underscores the potential of IL-6 as a prognostic biomarker for stroke severity and clinical outcomes, supporting its integration into patient monitoring and treatment planning.

Serpe et al. (contribution 10) summarized the evidence for blood-derived long non-coding RNAs (lncRNAs) as candidate biomarkers in autism spectrum disorder (ASD). Their review highlights lncRNAs linked to disrupted biological pathways in ASD and emphasizes the need for validation in larger, younger cohorts. Integrating multiple datasets and high-throughput approaches may ultimately enable early diagnosis and inform therapeutic strategies.

Hon and Naidu (contribution 11) provided an overview of metabolic reprogramming in the five most lethal cancers, identifying enzymes and transporters involved in glycolysis, amino acid metabolism, and lipid synthesis as potential biomarkers. While only a few markers, such as tumor M2-pyruvate kinase and serum LDH, are currently in clinical use, the review highlights the promise of metabolic biomarkers for early detection, prognosis, and treatment stratification, emphasizing the translational potential of these molecular indicators.

## 3. Conclusions

The articles in this Special Issue collectively illustrate the breadth and depth of contemporary biomarker research. From metabolomics and glycomics to immunoprofiling, non-coding RNA analysis, and cutting-edge sequencing technologies, these studies highlight how multidisciplinary approaches are driving innovation. They reinforce the crucial role of biomarkers in advancing early diagnosis, refining prognosis, guiding treatment selection, and monitoring disease evolution across diverse medical fields.

As technologies continue to evolve and integrative analytical methods become increasingly sophisticated, the discovery and validation of clinically meaningful biomarkers will accelerate further. Efforts to harmonize methodologies, enhance longitudinal study designs, and incorporate artificial intelligence will be essential to ensure that these advances translate into improved health outcomes. The contributions to this Special Issue reflect an exciting moment in molecular medicine and underscore the promise of biomarkers to reshape the future of personalized healthcare.
